# Analyses of the Binding between Water Soluble C60 Derivatives and Potential Drug Targets through a Molecular Docking Approach

**DOI:** 10.1371/journal.pone.0147761

**Published:** 2016-02-01

**Authors:** Muhammad Junaid, Eman Abdullah Almuqri, Junjun Liu, Houjin Zhang

**Affiliations:** 1 Department of Biotechnology, College of Life Science and Technology, Huazhong University of Science and Technology, Wuhan, Hubei, China; 2 Key Laboratory of Molecular Biophysics, Ministry of Education, Wuhan, Hubei, China; 3 Tongji School of Pharmacy, Huazhong University of Science and Technology, Wuhan, Hubei, China; Wake Forest University, UNITED STATES

## Abstract

Fullerene C60, a unique sphere-shaped molecule consisting of carbon, has been proved to have inhibitory effects on many diseases. However, the applications of C60 in medicine have been severely hindered by its complete insolubility in water and low solubility in almost all organic solvents. In this study, the water-soluble C60 derivatives and the C60 binding protein’s structures were collected from the literature. The selected proteins fall into several groups, including acetylcholinesterase, glutamate racemase, inosine monophosphate dehydrogenase, lumazine synthase, human estrogen receptor alpha, dihydrofolate reductase and N-myristoyltransferase. The C60 derivatives were docked into the binding sites in the proteins. The binding affinities of the C60 derivatives were calculated. The bindings between proteins and their known inhibitors or native ligands were also characterized in the same way. The results show that C60 derivatives form good interactions with the binding sites of different protein targets. In many cases, the binding affinities of C60 derivatives are better than those of known inhibitors and native ligands. This study demonstrates the interaction patterns of C60 derivatives and their binding partners, which will have good impact on the fullerene-based drug discovery.

## Introduction

The C60 molecule (fullerene) was first discovered during the laser irradiation of graphite [1] and then produced by graphite vaporization through ohmic heating [2]. Since its discovery, fullerene C60 has gained much attention due to its broad applications in many fields[3, 4].The early research focused on the physical properties of C60 molecule, while many methods for its functionalization were developed later[5], such as halogenation[6], hydrogenation[7], epoxidation[8] and alkylation[9]. Using these techniques, a number of C60-based compounds have been synthesized, which have shown promising effects on various diseases [10, 11].

The main hindrance of C60 application in medicine is the lack of solubility in polar solvents and the formation of aggregates in aqueous solutions. The native C60 molecule has a limited application in biomedical research because it is only soluble in organic solvents. In response to this limitation, functionalized water soluble C60 molecules, such as polyhydroxylated C60, was first synthesized in 1992 [12]. By studying the quantitative structure solubility relationships of C60 with 75 organic solvents, different methods have been developed to overcome the solubility problem of C60 molecules [13]. C60 molecule can be covalently linked to cyclodextrin and calixarenes to enhance its solubility [14, 15]. Other methods such as the use of the detergent Tween-20 [16],micelles [17, 18], polyvinylpyrrolidone [19], liposomes [17], phospholipids [20], vesicles [18] are also used to enhance its solubility.

The pioneering work for the application of C60 derivatives in medical uses was carried out by Friedman *et al* [21]. Friedman *et al*. noticed that the radius of C60 and HIV protease (HIVP) active site are almost the same and the nature of both of C60 and HIVP active site are hydrophobic. Thus, hydrophobic interactions may exist between C60 and HIVP active site. The computational study indicates that the formation of HIVP/C60-derivatives complex will remove 298 Å^2^ solvent exposed area. The primary hydrophobic nature of this area will provide the driving force for the complex formation. To investigate this interaction, bis(phenethy1amincuccinate)C60, a derivative of C60 molecule, was synthesized by R. Sijbesma *et al*. [22]. The pharmacological evaluation shows that this C60-derivative has a good inhibitory activity with EC50 7μM against HIV-1 infected human peripheral blood mononuclear cells[21]. The kinetic study indicates that the derivative will inhibit the binding of the natural substrate of HIVP through a competitive mode. Its inhibitory activity was found to be due to the attachment of polar functional group such as cationic moieties[21]. In addition, many other C60 derivatives were synthesized and proven to have good inhibitory effects against different targets. For example, the C60 derivative, fullerene-carboxylic acid, was reported to act as a selective DNA cleavage agent at guanine base under photoirradiation[23]. This kind of derivative has also been shown to inhibit the excitotoxic death of cultured cortical neurons. It can delay the death and functional deterioration of the transgenic mouse carrying the mutant gene for familial amyotrophic lateral sclerosis[24]. The other derivative, polyhydroxylated C60, acts as a free-radical scavenger [25]. It has been shown to prevent the hydrogen peroxide or cumene hydroperoxide-elicited reduction of the population spikes in the hippocampal slices. Other research showed that the polyhydroxylated C60 can function as the neuroprotective agents in cortical cell cultures exposed to excitotoxic and apoptotic injuries. It can reduced neuronal apoptosis caused by serum deprivation, suggesting it is an anti-oxidant compound [26]. The possible antiproliferative mechanism of this compound was studied using rat vascular smooth muscle cells (A7r5 cells), which indicates that it inhibited the proliferative responses in a concentration dependent manner. This inhibition mechanism is partly mediated through the inhibition of the protein tyrosine kinase [27]. Also, Jin, H *et al*. have shown that polyhydroxylated C60 are effective in blocking glutamate induced neurotoxicity especially due to their antagonistic effects on the glutamate receptors and the effects of lowering the intracellular calcium concentration. The inhibition can be achieved at a dose-dependent fashion with IC50 of 50 microM [28]. Recently, it has been shown that the sodium fullerenolate Na4[C60(OH)~30] (NaFL) can destroy amyloid fibrils of the Ab(1–42) peptide in the brain. In the *in vitro* test, this C60 derivative can also prevent the formation of amyloid fibrils. Meanwhile the *in vivo* test shows very mild toxicity. The maximal tolerable dose (MTD) and LD50 are only 1000 mg kg-1 and 1800 mg kg-1 respectively [29]. Its free radical scavenging property also leads to the protective effects on decreases in mitochondrial function, increases in the levels of reactive oxygen species, elevated oxidative damage to DNA/proteins, and MPP-induced loss in cell viability[30].

Although it has been demonstrated that the derivatives of C60 molecule have inhibitory effects on many drug targets, the patterns with which C60 derivatives may interact with the target proteins have rarely been studied. In the present study, a database of water soluble C60 derivatives was constructed with their chemical structures published in the literature. The water soluble C60 derivatives were docked with the C60-binding proteins reported in the literature[31]. The binding affinities of water soluble C60 derivatives with various drug targets were investigated using computational tools. Subsequent analyses indicate that water soluble derivatives of C60 have better binding affinities with target proteins than the known inhibitors or native ligands. Therefore, these water soluble derivatives may serve as the lead compounds for fullerene-based drug discovery.

## Materials and Methods

### Preparation of C60 derivatives

A literature search was performed to collect chemical structures of water soluble C60 derivatives reported by different laboratories (S1 Table) [32–37]. The Builder module embedded in MOE2014 was used to draw the three-dimensional structures of the C60 derivatives, which were used to construct a ligand database. Partial charges were calculated for all the molecules using the Merck Molecular Force Field 94X (MMFF94X), which is suitable for small molecules [38]. Subsequently, the energy of all C60 derivatives was minimized with a RMS gradient of 0.05 kcal/mol Å^2^.

### Molecular Docking

MOE-Dock program embedded in MOE2014 was used for docking. The crystal structures of the target proteins were retrieved from the protein data bank (PDB). The bound water molecules in each crystal structure were removed. The structural preparation program embedded in MOE was used to add any missing hydrogen atom, correct the charges and assign correct hybridization state of each residue. The protonate 3D module embedded in MOE was used to assign the correct protonation state using Generalized Born/Volume Integral (GB/VI) electrostatic function. The whole structure of each enzyme was used as a receptor to find the potential binding sites. Multiple conformations were generated for each ligand by applying a preferred torsion angles to all rotatable bonds in each ligand. Thirty conformations were generated for each C60 derivative. The accepted conformations for each ligand against each receptor were scored using London dG scoring function which calculates the free energy for the binding of ligand from a given conformation.

All the accepted complex conformations were submitted to a further refinement step, based on the force field Amber12: EHT. The side chains of the receptor proteins were held fixed during the refinement. The GBVI/WSA dG scoring function along with the Generalized Born solvation model (GBVI) was used to calculate the final energy and docking score [39]. The Amber12: EHT force field was used for all the computational procedures.

### Binding Affinity Calculations

Binding affinities of all the water soluble C60 derivatives were calculated using Generalized-Born Volume Integral/Weighted Surface area (GBVI/WSA) method embedded in MOE. Generalized Born interaction energy is the non-bonded interaction energy between the receptor molecule and the ligand. It comprises Coulomb electrostatic interaction, Van der Waals, and implicit solvent interaction energies [40]. During calculation the atoms of the receptors 8 Å away from the ligand were kept fixed. The atoms of the receptor that is in vicinity of the ligand were made flexible but were subjected to the tether constraint with a force constant of 10 that discourage the gross movement. No tether restraint was applied to the ligand atoms. In each case an energy minimization of binding pocket in each C60 derivative complex was performed before calculating binding affinity. The binding affinity was calculated for each hit after energy minimization, and reported in unit of kcal/mol.

### Molecular Dynamics Simulations

The complexes having the good binding affinities were subjected to the molecular dynamics (MD) simulations using the Amber14 software. The MD simulations were carried out in order to check the stability of each derivative in the active site of each enzyme. The *ff14SB* force field was used to define the protein using the *tleap* module of AmberTools15 [41]. *tleap* was also used to add hydrogen atoms to each complex and neutralize each system with Na+ counter ions. Each system was then immersed into the rectangular box of TIP3P water molecule with a buffer distance of 8 Å [42]. The accelerated GPU *pmemd* code was used to perform all steps of MD for each system [43]. The minimization of each system was done in six steps including 1000 steps of steepest descent minimization followed by 1000 steps of conjugate gradient minimization at each step. Initially, the positional restraints on water molecules were kept 500kcal/mol/Å^2^ and systematically lowered down to zero in six steps. After minimization, each system was heated up to 300K in 1 nanosecond (ns) through five steps. Initially the positional restraints on water molecules during heating were kept 500kcal/mol/Å^2^ and systematically lowered down to 100kcal/mol/Å^2^ in five steps. The heating was followed by density equilibration step. The density of each system was equilibrated with weak restraints for the 5ns followed by the equilibration of the whole system at constant pressure for the 5ns. Finally, the whole system was subjected to unrestrained MD simulation for the 25 ns, saving the trajectory after each 20 ps. During simulation the pressure was kept constant and temperature was control with Langevin thermostat (1 atm, 300K) [44]. Long-range electrostatic interactions were computed by employing Particle Mesh Ewald (PME) with the default setting in AMBER14 [45, 46]. The cutoff distances for the long range electrostatic and van der Waals interactions were set to 10.0 Å. The SHAKE algorithm was used for the covalent bonds involving hydrogen [47]. The trajectory of MD simulation was analyzed for the structure stability.

### Tunnel Prediction and Visualization

The program CAVER 3.0 was used for of access tunnel computation [48]. The X-ray structures were used as input files for CAVER to find potential access tunnels involved in ligand egress. All the structures and access tunnels presented in this paper were visualized and rendered in PyMol 1.6. The sphere representation was used for all the access tunnels and C60 derivatives. The sphere scale was set to 1.

## Results and Discussion

Carbon atoms can form a number of stable nanostructures by arranging into different geometries. A graphene can be shaped into a single walled nanotube with a diameter of 1 nanometer (nm), while carbon atoms can also be arranged into spherical structures such as fullerenes. Among various fullerenes, C60 molecule is the most stable and readily available with a diameter of 0.72 nm. Based on the original C60 molecule, numerous water soluble C60 derivatives have been synthesized by other researchers, with the intention to explore the additional functions of C60 molecules (S1 Table)[32–37]. To assay whether water soluble C60 derivatives can interact with different C60-binding proteins, the predicted C60-binding proteins were classified into several groups. The representatives of each group were used to perform a molecular docking study with the C60 derivatives. The interactions between C60 derivatives and target proteins were quantified in term of binding affinities. The binding of C60 was compared to the interaction of the target proteins and their known inhibitors. Interestingly, several C60 derivatives have better affinities, which underline their potential to be developed as the novel inhibitors of these proteins.

### Acetylcholinesterase

**Fig 1:**
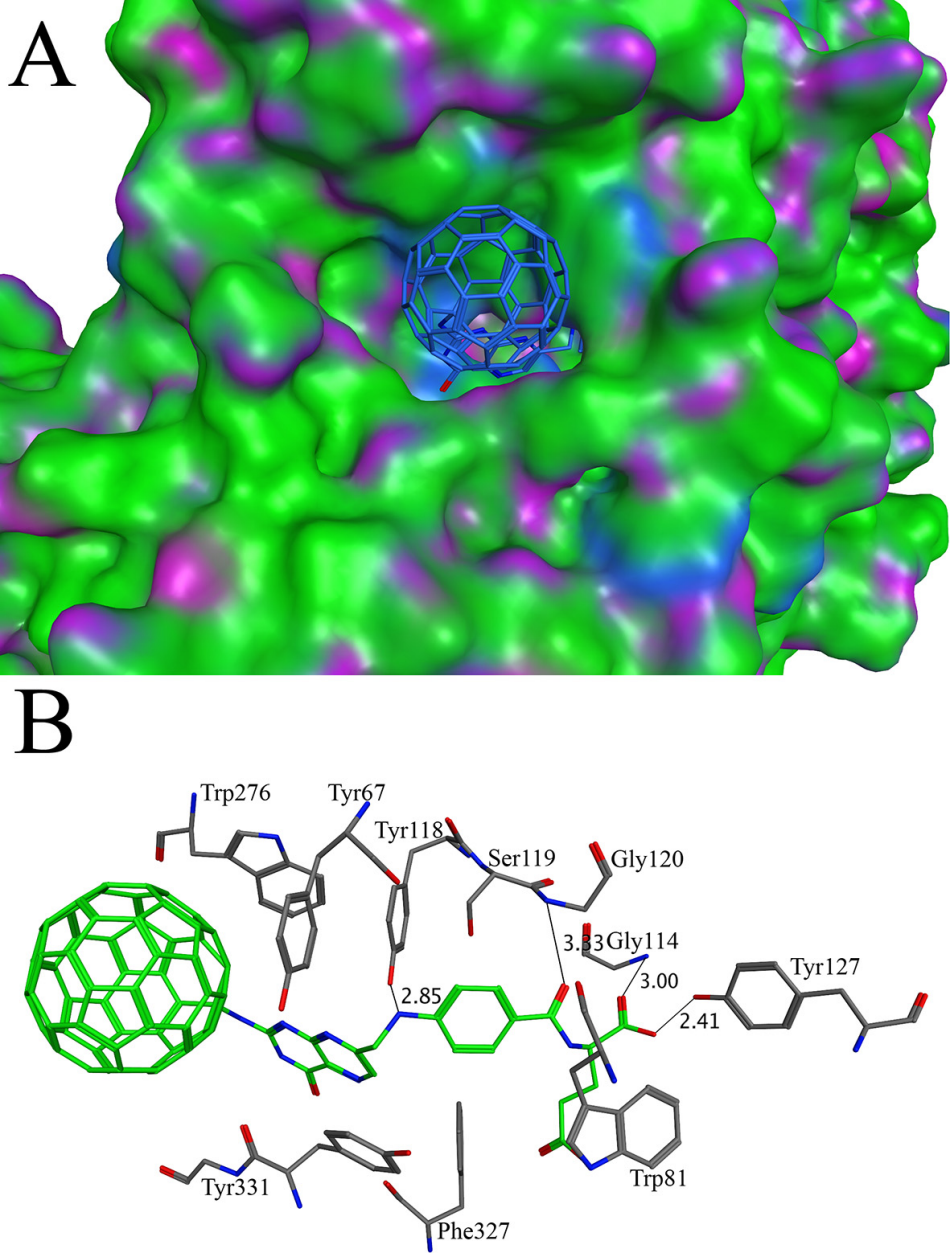
Docking of C60-16 with acetylcholinesterase: (A) The binding of C60-16 in the active site of acetylcholinesterase. The C60-16 is shown in stick model while the surface of acetylcholinesterase is shown in green, blue and purple (B) The three dimensional analysis of the interactions of C60-16 in the active site of acetylcholinesterase. The C60-16 makes interactions with Tyr67, Trp81, Gly114, Tyr118, Ser119, Gly120, Tyr127, Trp276, Phe327 and Tyr331.

Alzheimer’s disease, the most common cause of dementia, accounts for 60–80% of the total cases. In United States, one in every eight persons is suffering from Alzheimer’s disease, making it sixth leading cause of death in United States [49, 50]. Acetylcholinesterase, a serine protease, is found at the neuromuscular junctions and cholinergic brain synapses. It hydrolyzes the neurotransmitter, which releases choline and acetyl group as product and terminates the relaying signal [51, 52]. The activity of this enzyme increases in Alzheimer’s disease patients, making it a promising target to be inhibited for the treatment of the disease. The X-ray crystal structure of acetylcholinesterase (PDB ID 1ACJ) in complex with tacrine was used as a receptor for the docking calculation. The docking of C60 derivatives was carried out without defining the active site. A potential binding pocket was found present at the bottom of a 20 Å gorge at the center of the molecule. The four FDA approved drugs donepezil [53, 54], rivastigmine [55, 56] tacrine [57] and galantamine [58] targeting acetylcholinesterase were docked using the same method as C60 derivatives. They are found to bind at the same pocket. The binding affinities of the FDA approved drugs are listed in S2 Table. As compared to the values in the S2 Table for the FDA approved drugs, only one C60 derivative C60-16 has a better binding affinity. The C60-16 binds tightly in the active site with a binding affinity of -14.44 kcal/mol (Fig 1A). The binding of the C60-16 at the active site involves the residues that span the whole length of the active site gorge. The aromatic rings of the Trp276 and Tyr67 stack against the aromatic rings of C60-16, making π-π interactions at the entrance of the active site (Fig 1B). The residues Trp81, Phe327 and Tyr331 are involve in the C-H/Pi interaction. The orientations of Trp276 and Trp81 are in consistence with the previous study of the Jonah Cheung *et*.*al*. [54] and they are involved in the hydrophobic interaction. A direct hydrogen bond is formed between the OH of the Tyr118 and the N2 of the C60-16. In addition, hydrogen bonds deep at the active site gorge are formed between the C60-16 and Gly120, Gly114 and Tyr127. Recently, the prediction of C60 derivative’s binding site in acetylcholinesterase is reported by the Arlan da Silva Gonçalves *et*. *al* [34]. Our docking studies of soluble C60 derivatives with acetylcholinesterase are in consistence with the study of Arlan da Silva Gonçalves, as these C60 derivatives docked into the same binding site. In addition, the access tunnel prediction based on the crystal structure found two potential routes. As shown in the S1 Fig, the C60-16 blocks the access tunnel A completely while it partly blocks the access tunnel B. It is noteworthy that the active site of the acetylcholinesterase is buried deep in a cavity on the protein surface. As it is partially shielded from the water molecules outside, the wall of the active site is covered with large aromatic residues. As indicated by the earlier structural study, these aromatic residues provide significant binding force for the protein-ligand complex [59]. It is no surprise that the C60 derivative with an aromatic side show better binding affinity than the original ligands. Due to the narrow configuration of the active site, the derivative with a long side chain is required for the binding. On the other side, the long side chain pushes the C60 moiety away from the binding site, and the C60 is well exposed on the surface of the protein. Therefore, a shorten side chain of the same derivative may have a better binding affinity with the target protein.

### Glutamate racemase

**Fig 2:**
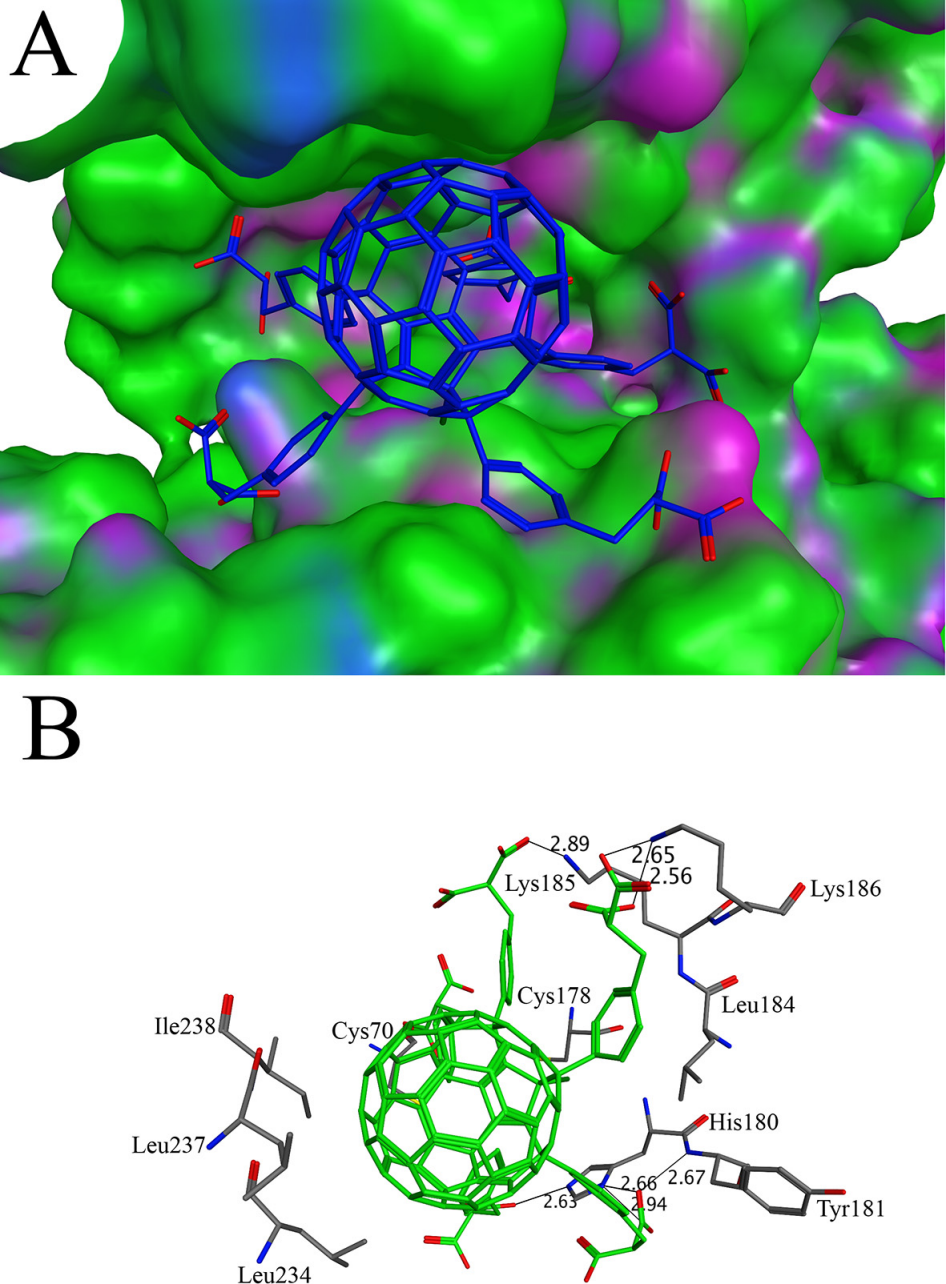
Docking of C60-11 with glutamate racemase: (A) The binding of C60-11 in the active site of glutamate racemase. The C60-11 is shown in stick model while glutamate racemase is shown in surface model (B) The interactions between C60-11 and glutamate racemase. The C60-11 makes interactions with Cys70, Cys178, His180, Tyr181, Leu184, Lys185, Lys186, Leu234, Leu237 and Ile238. The distances between interacting atoms are shown in angstroms.

The peptidoglycan layer is unique to bacteria. The enzymes involved in the synthesis of peptidoglycan layer have been considered as the target of many antibacterial drugs [60, 61]. The D-amino acids in peptidoglycan protect the cell wall from the proteases [61]. Glutamate racemase is one of the essential enzymes that convert the L-glutamate to the D-glutamate. It has been used as drug target for the designing of new antibiotics [61, 62]. The crystal structure of glutamate racemase (PDB ID 1B74) in complex with substrate analog, D-glutamine, was used for the docking. The docking of C60 derivatives and known inhibitors, compounds C1-8 (S3 Table)[63], were carried out without defining the active site. The binding affinities of already published inhibitors were given in S3 Table. As compared to the values in the S3 Table, only one C60 derivative C60-11 has a good binding affinity. The C60-11 binds tightly in the active site with a binding affinity of -13.79 kcal/mol (Fig 2A). Due to the bulky side groups of the C60-11, it makes several hydrogen bonds with active site residues Lys185, Lys186, His180 and Tyr181 (Fig 2B). The previous biochemical studies identified the substrate binding site in the deep pocket. It has been shown in the biochemical study that the residues Cys70 and Cys178 are important for the catalysis [64–66]. For the stereo-inversion of L-glutamate to D-glutamate, the Cα of the L-glutamate should be within the close distance to the Cys70 and Cys178 for the deprotonation and protonation. The Cys70 and Cys178 residues lie at the bottom of the active site (Fig 2B). The binding of C60-11 blocks the access to these residues and could be a potent inhibitor of glutamate racemase. The access tunnel prediction shows only one potential route that leads to the active site. C60-11 completely blocks the access tunnel (S1 Fig). Glutamate racemase exists in dimer form. The dimerization is important for the stability of the glutamate racemase. The binding site of the C60-11 overlaps with the dimeric interface of the glutamate racemase. The side chain of the residues Leu184, Leu234, Leu237 and Ile238 are found to have hydrophobic interaction with the C60-11 (Fig 2B). As these residues are important for the dimerization [67], the C60-11 may also serve the function of inhibiting the enzyme by preventing the formation of the dimer.

### Inosine monophosphate dehydrogenase

**Fig 3:**
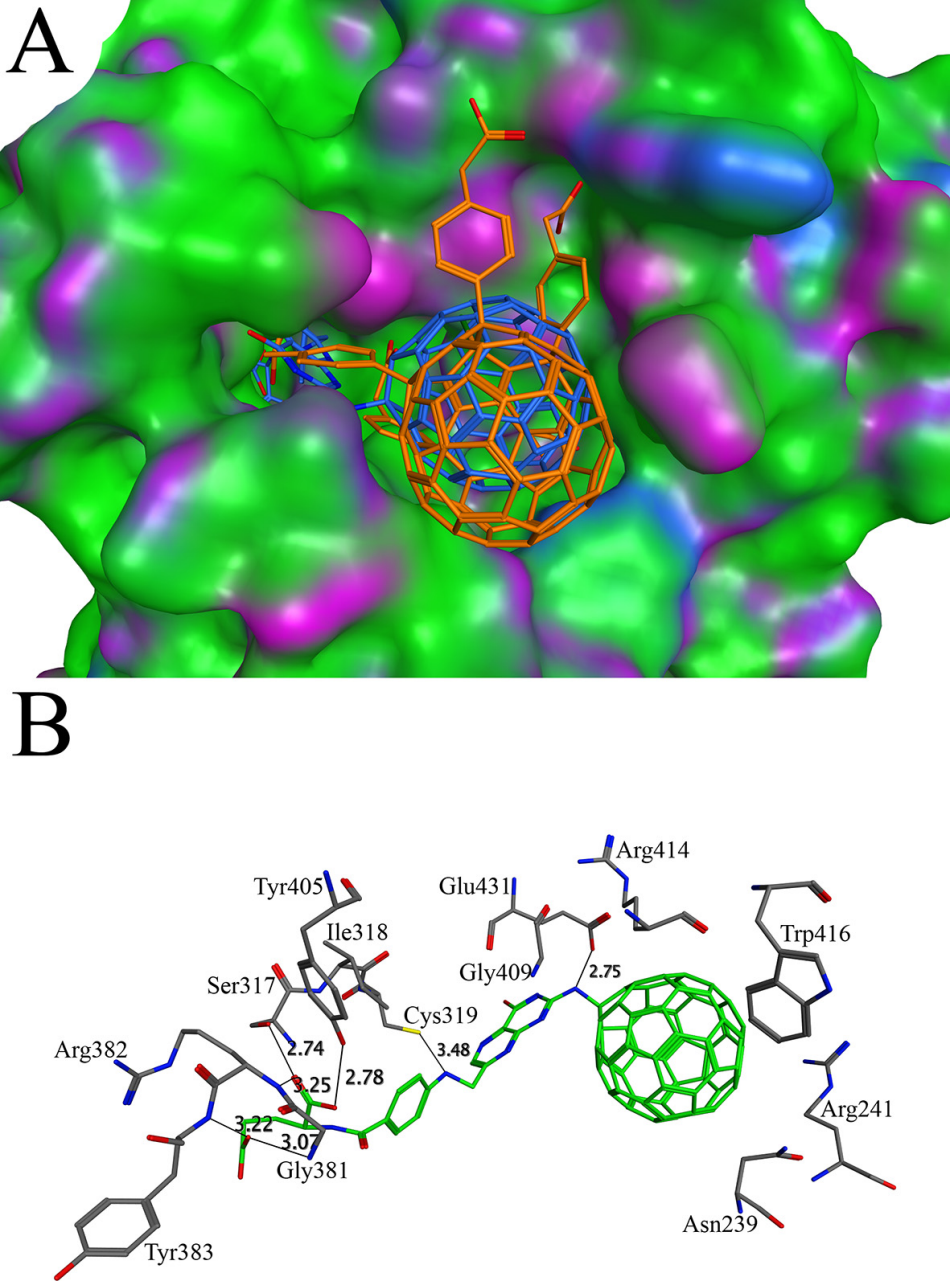
Docking of C60-10 and C60-16 with inosine monophosphate dehydrogenase: (A) The binding patterns of C60-10 and C60-16 in the active site of inosine monophosphate dehydrogenase. The C60-10 and C60-16 are shown in stick model with golden and blue color respectively while the surface of inosine monophosphate dehydrogenase is shown in green, blue and purple (B) The analysis of interactions of C60-16 in the active site of inosine monophosphate dehydrogenase. The C60-16 makes contacts with Asn239, Arg241, Ser317, Ile318, Cys319, Gly381, Arg382, Tyr383, Tyr405, Gly409, Arg414, Trp416 and Glu431. The contacts are marked as lines and the distances between interacting atoms are shown in angstroms.

Tuberculosis is a pandemic disease, effecting nearly two billion people worldwide. The pathogenic agent of this contagious infectious disease has been proved to be the *Mycobacterium tuberculosis* [68]. Inosine monophosphate dehydrogenase (IMPDH) is the key enzyme, controlling the rate limiting step in the guanine nucleotide biosynthesis. It converts inosine monophosphate to xanthosine monophosphate in a NAD^+^-dependent reaction. The IMPDHs from many species have been characterized and used as drug target[69]. The X-ray crystal structure of IMPDH in complex with the ribavirin (PDB ID 1ME8) was used for the docking purpose. The binding affinity of the ribavirin was calculated as -9.97 kcal/mol and used as a cutoff value to select C60 derivatives with good affinities. Two of the C60 derivatives C60-16 and C60-10 have better binding affinities than ribavirin. The binding affinities of the C60-16 and C60-10 were found to be -15.00 kcal/mol and -13.63 kcal/mol respectively. The cavity of the binding site, found by the MOE software, is narrow and long, showing consistency with the binding pocket in the IMPDH/ ribavirin complex crystal structure [70]. The side group attached to the C60-16 is long enough to reach the bottom of the active site (Fig 3A). In case of C60-10, several short side groups are attached to the C60 molecule. Due to the bulky groups, the C60-10 binds to the surface cavity instead binding deep in the pocket. The dissimilarity between the side groups on C60-16 and C60-10 may account for the difference in the binding affinity. The C60-16 makes several hydrogen bonds along with hydrophobic interactions with the active site residues (Fig 3B). The perpendicular orientation of the indole group of Trp416 to the aromatic ring of the C60-16 leads to C-H/Pi interaction. As discussed previously, the C-H/Pi interactions are stable both in polar and non-polar solvents. Hence, these interactions have a good contribution to the stability of complex [71]. The hydrophobic interaction was found between the Ile318 and aromatic ring of C60-16 side group. The NH/pi interaction was also found between the C60-16 and Asn239, Arg241 and Arg414. Several hydrogen bonds were observed between the C60-16 side group and Ser317, Gly381, Arg382, Tyr383 and Tyr405 deep at the bottom of the active site. The direct hydrogen bond also exists between the Cys319 and C60-16 involving the S-H group and N-H group respectively. The reduced sulfur is capable for donating or accepting hydrogen bonds [72]. The sulfhydryl group of Cys319 forms hydrogen bond with the N2 of the C60-16. As compared to other hydrogen bonds, the distance between Cys319 and C60-16 is longer (3.48 Å). The equilibrium distance between hydrogen bond donor and acceptor involving sulfur is longer than oxygen and hydroxyl group due to the larger size of the sulfur and more diffuse electron cloud [72].

### Lumazine synthase

**Fig 4:**
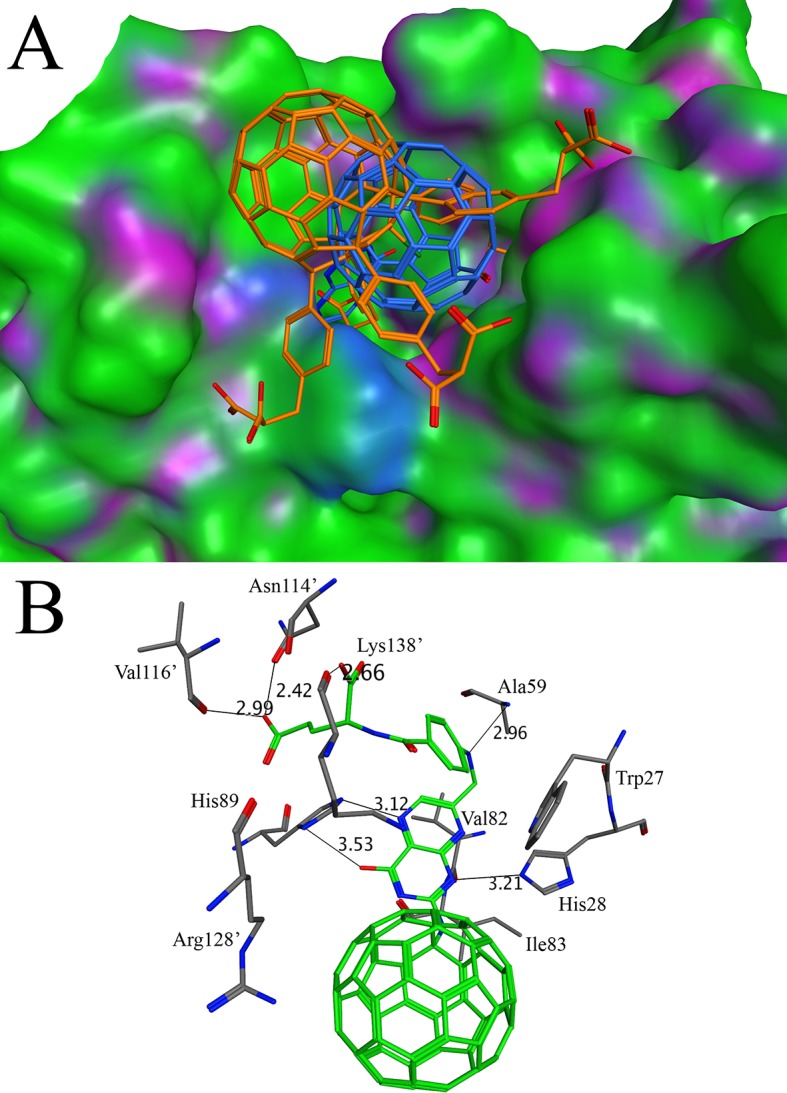
Docking of C60-16 and C60-11 with lumazine synthase: (A) The binding of C60-16 and C60-11 in the active site of lumazine synthase. The C60-16 and C60-11 are shown in sticks with blue and golden color respectively while the surface of lumazine synthase is shown in green, blue and purple (B) The interactions of C60-16 in the active site of lumazine synthase. The C60-16 makes interactions with the resides from chain A Asn114’, Val116’, Arg128’, Lys138’ and from chain B Trp27, His28, Ala59, His89, Val82, Ile83. The distances of various atoms are marked as thin lines and shown in angstroms.

Riboflavin, a water soluble B vitamins, plays a significant role in the metabolic reactions. The source of riboflavin in the human is food because the enzymes for the biosynthesis of riboflavin are absent in human. Most of the pathogenic organisms depend on the endogenous biosynthetic pathway for the riboflavin because the microorganisms are unable to take riboflavin from the diet[73]. Lumazine synthase is the enzyme that catalyzes the last step of riboflavin synthetic pathway. Due to the fact that this enzyme is absent in human, it represents an attractive target for the development of new drug against pathogenic organisms[74]. The X-ray crystal structure of lumazine synthase in complex with inhibitor TS51 (PDB ID 2C9D) was used for the docking purpose [75]. The binding affinity of the native inhibitor was found to be -13.66 kcal/mol. Out of 16 derivatives, only two derivatives C60-16 and C60-11 have better binding affinities than TS51. The binding affinities of C60-16 and C60-11 were found to be -15.37 and -14.64 respectively. The side groups of both derivatives fit well in the active site of lumazine synthase (Fig 4A). The binding site for the C60-16 and C60-11 is same as described for the other previously reported inhibitors [76]. The binding site is present at the interface and involves residues from the two subunits of lumazine synthase. The importance of key residues (Arg127, His88, Lys135), corresponding to Arg128, His89 and Lys138 in the present study, has been discussed by Zhang, X *et*, *al* [77]. The His89 and Lys138 make hydrogen bonds with C60-16 while Arg128 is involved in the NH/Pi bond (Fig 4B). His89 is conserved in all known lumazine synthase and is suggested to be involved in proton transfer due to its location in the vicinity of substrate [77]. The mutation of this His residue reduces the activity of the enzyme which implies its importance in the catalytic activity [78]. The binding of C60-16 disturbs the position of His89 by 90 degrees, which may disturb the proton transfer. Additionally, the hydrogen bonds are also observed that involve the C60-16 and His28, Ala59, Asn114 and Val116. The binding of C60-16 imposes change in the orientation of Trp27 side chain. As discussed in previous studies, the Trp27 have two conformations with two different inhibitors related to each other by rotation of 180 degree making either π-π interaction or C-H/Pi interaction [75]. In case of the present study, the indole group of Trp27 is perpendicular to the aromatic ring C60-16 molecule making C-H/Pi bond. The C-H/Pi bond is also observed with Val82 and Ile83 (Fig 4B).

### Human estrogen receptor alpha

**Fig 5:**
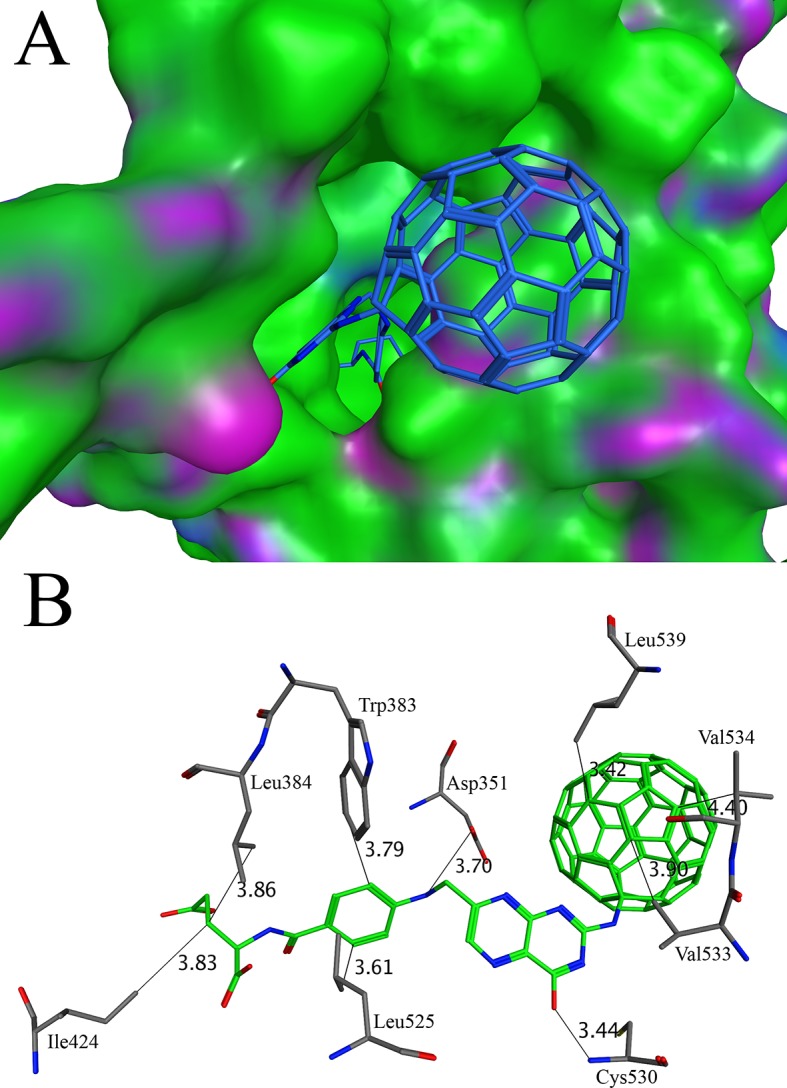
Docking of C60-16 with human estrogen receptor alpha: (A) The binding of C60-16 in the active site of human estrogen receptor alpha. The C60-16 is shown in stick while the surface of human estrogen receptor alpha is shown in green, blue and purple (B) The three dimensional analysis of the interactions of C60-16 in the active site of human estrogen receptor alpha. The C60-16 makes interactions with Asp351, Leu384, Trp383, Ile424, Leu525, Cys530, Val533, Val534 and Leu539. The distances between various atoms are marked as thin lines.

The steroid hormones receptors are a large group of proteins, including estrogen receptor α. At present, approximately 70% of the breast cancer is positive for estrogen receptor expression [79]. It has been proved that estrogen sustains the growth of cancer cell because it expresses the receptors for the estrogen. Hence, it is established that estrogen is involved in the pathogenesis of breast cancer [79]. The hormonal therapy is required to treat breast cancer that aims to block the stimulation of estrogen in breast cancer cells. It has been achieved through the blocking of estrogen receptor α [80]. The screening conducted with the structure of estrogen receptor α (PDB ID 3ERT) indicates that C60-16 has better affinity with this receptor. The affinities of C60-16 and native antagonist 4-hydroxytamoxifen (tamoxifen) were found to be -11.06 kcal/mol and -8.47 kcal/mol respectively. The C60-16 binds to the same pocket that recognizes the agonist diethylstilbestrol, antagonist 4-hydroxytamoxifen and raloxifene [81]. The C60-16 has a long side group and fit well in the active pocket (Fig 5A). Near the entrance of the active site, the Asp351 is involved in a hydrogen bond (Fig 5B). C60-16 also makes hydrogen bond with Cys530. At the entrance of the active site the C60-16 has a hydrophobic interaction with the residues Val533, Val534 and Leu539. At the bottom of the active site, the aromatic ring of C60-16 mimic the C ring of the 4-hydroxytamoxifen [80] and make hydrophobic interactions with the residues Trp383, Leu384, Ile424 and Leu525. It is noteworthy that the inhibitor embedded in the complex crystal structure has a several aromatic rings. Through the hydrogen bonds and hydrophobic interactions, the aromatic systems in the C60 moiety as well as the side chain provide a good portion of the binding force.

### Dihydrofolate reductase

**Fig 6:**
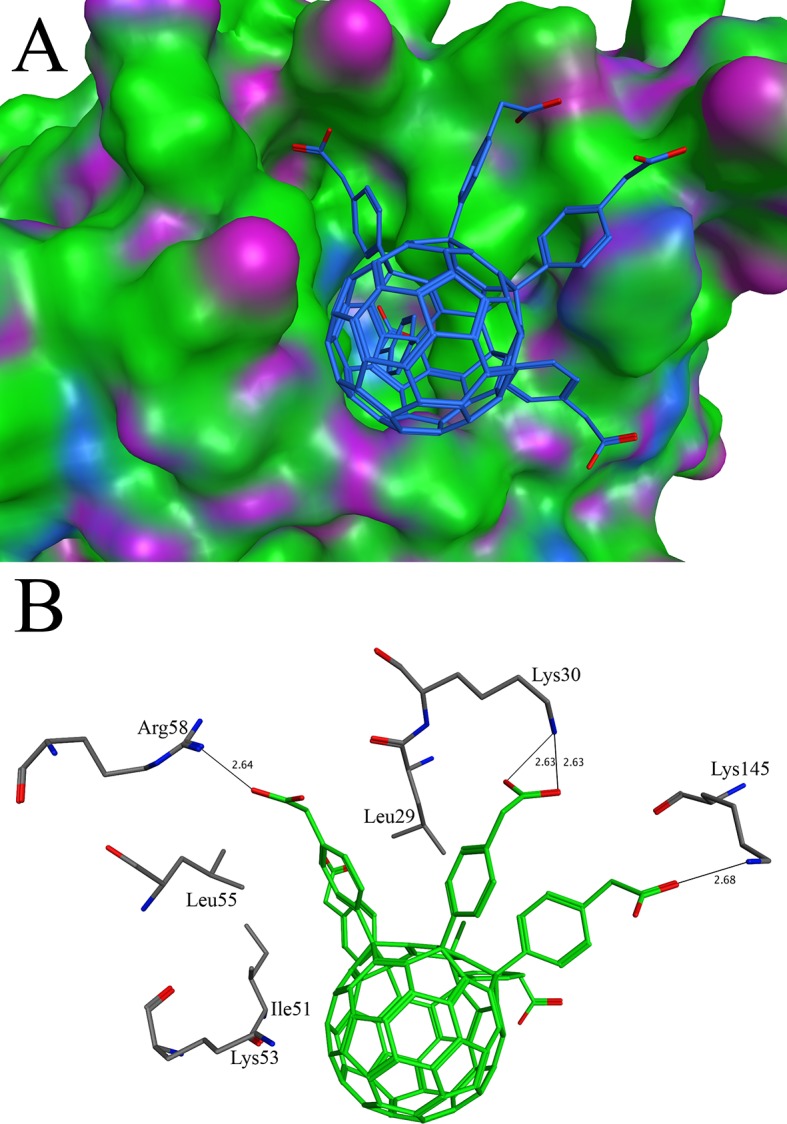
Docking of C60-10 with dihydrofolate reductase: (A) The binding pattern of C60-10 in the active site of dihydrofolate reductase. The C60-10 is shown in stick while dihydrofolate reductase is shown in surface model (B) The diagram of the interactions bewteeen C60-10 and dihydrofolate reductase. The C60-10 makes interactions with Leu29, Lys30, Ile51, Lys53, Leu55, Arg58 and Lys145. The hydrogen bonds are shown in thin lines.

Dihydrofolate reductase catalyzes the NADPH dependent reduction of dihydrofolate to tetrahydrofolate. It is an important enzyme in the purine and thymidine biosynthetic pathway. It has been proved as a good drug target [82]. The molecules that target this enzyme are shown to have a potential antibiotic ability [83]. Our docking study of C60 derivatives conducted with the X-ray crystal structure of dihydrofolate reductase (PDB ID 4LAE) shows that C60-10 has a good binding affinity. The binding affinities of C60-10 and native ligand were found to be -12.29 and -10.59 respectively. The binding of C60-10 is shown in the Fig 6A. One of the side groups of the C60-10 fills a hydrophobic pocket, which mimics the position of 5,6-dimethylbenzimidazole ring of the native ligand [84]. The aromatic ring of the C60-10 is sandwich between the Leu29 and Leu55 that make the hydrophobic pocket (Fig 6B). This interaction is further strengthened by ionic bond of the same side group with Arg58. Other hydrogen bonds are also observed that contribute to the stability of C60-10 in the pocket. At the entrance of the active site, the C60-10 interacts with the side chain of Lys145 through hydrogen bond. In addition, the hydrogen bond also occurs between the side chain of Lys30 and C60-10 at the entrance of the active site. The core of the C60-10 makes several C-H/Pi interactions with Ile51. The Lys53 also make cation-π interaction with the C60-10. The cation-π interaction has many applications in the drug designing. The most stable interacting distance between the cation and aromatic ring is 2.5 to 3.5 Å [85]. In case of the present study, the distance between the Lys53 and C60-10 aromatic ring is 3.39 Å showing a strong interaction. In addition, the access tunnel used for transportation of substrate/product was predicted with the software CAVER[86]. The access tunnel prediction shows only one potential route that lead to the active site. The C60-10 is found to block the access tunnel completely (S1 Fig).

### N-Myristoyltransferase

**Fig 7:**
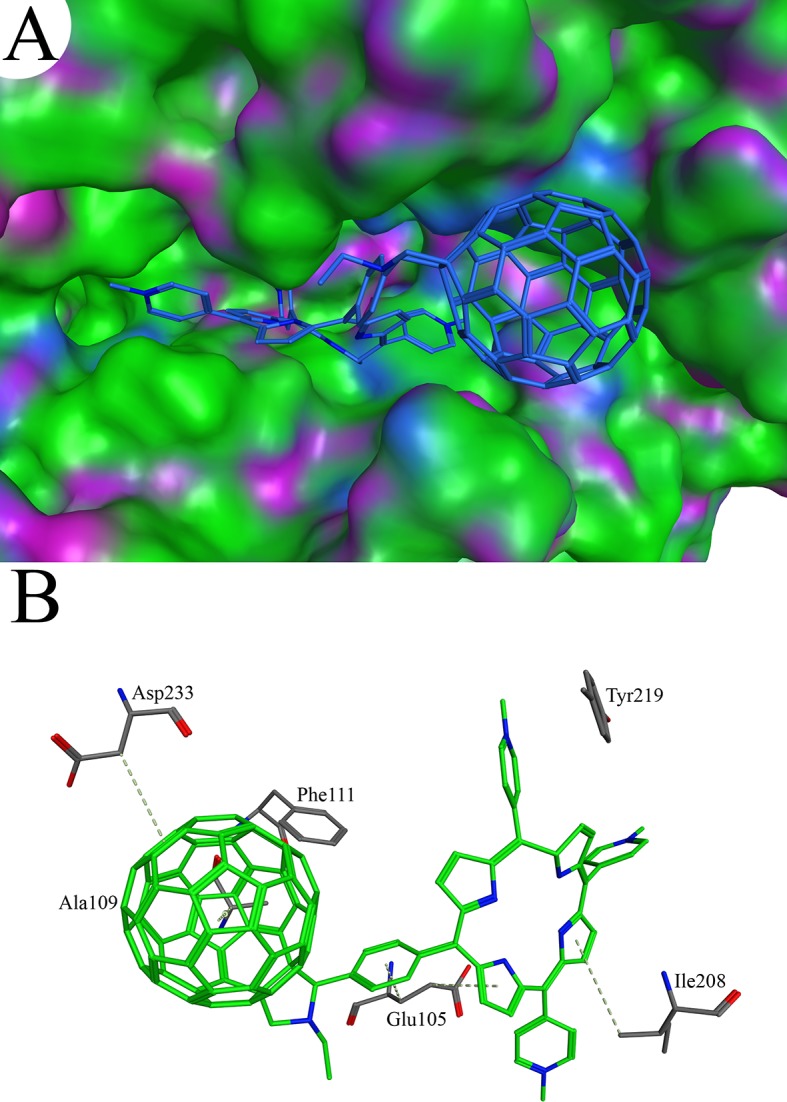
Docking of C60-12 with N-Myristoyltransferase: (A) The binding of C60-12 in the active site of N-Myristoyltransferase. The C60-12 is shown in stick model while the surface of N-Myristoyltransferase is shown in green, blue and purple (B) The analysis of the interactions between C60-12 and N-Myristoyltransferase. The C60-12 makes interactions with Glu105, Ala109, Ile208, Phe111, Tyr219 and Asp233.

The N-myristoylation, catalyzed by N-Myristoyltransferase, of the large proteins is important for the various cellular activities [87]. The N-Myristoyltransferase catalyzes the transfer of myristate group from the Co-enzyme (myristoyl-CoA) to the N-terminal glycine of eukaryotic and viral proteins [88]. The N-Myristoyltransferase has been proved as a drug target for many diseases such as leishmaniasis, malaria and Chagas disease [89–91]. The X-ray crystal structure of N-Myristoyltransferase with PDB ID 2P6F was used for the docking purpose. The screening conducted with the water soluble C60 derivatives indicates that C60-12 has a good binding affinity of -14.59 kcal/mol. This binding affinity is better than the native ligand (-5.33 kcal.mol). The binding profile of the C60-12 shows that it occupies the same binding site discussed in previous studies (Fig 7A) [92]. The C60-12 is stabilized by numerous hydrophobic interactions in the active site (Fig 7B). The Glu105, Ile208, Ala109 and Asp233 form C-H/Pi bond with C60-12. The orientation of the Phe111 is perpendicular to the aromatic ring of C60-12 and makes hydrophobic interaction. The aromatic ring of the Tyr219 is parallel to the aromatic ring of the 60–12 and makes parallel π-π interaction. The access tunnel prediction performed with CAVER shows three potential routes access tunnel A, B and C that lead to the active site. The side group of the C60-12 is large enough so that it may block the active site that is accessed by the three routes (S1 Fig).

### Molecular Dynamics Simulation

**Fig 8:**
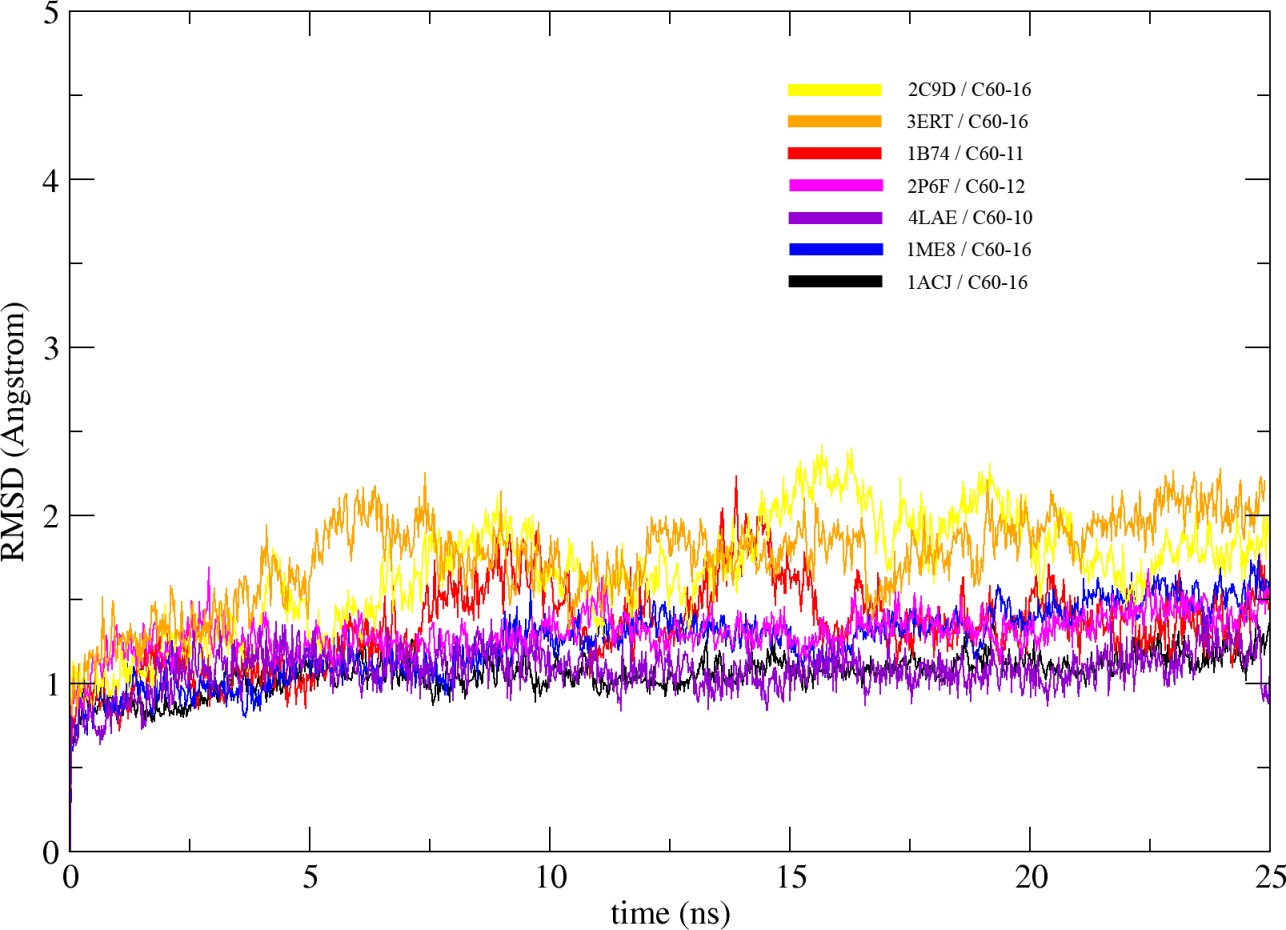
The molecular dynamics simulation of the C60 derivative/protein complexes: All the complexes are simulated over a period of 25 ns. The RMSD of the simulation was plotted against time, according to the simulation trajectories.

**Fig 9:**
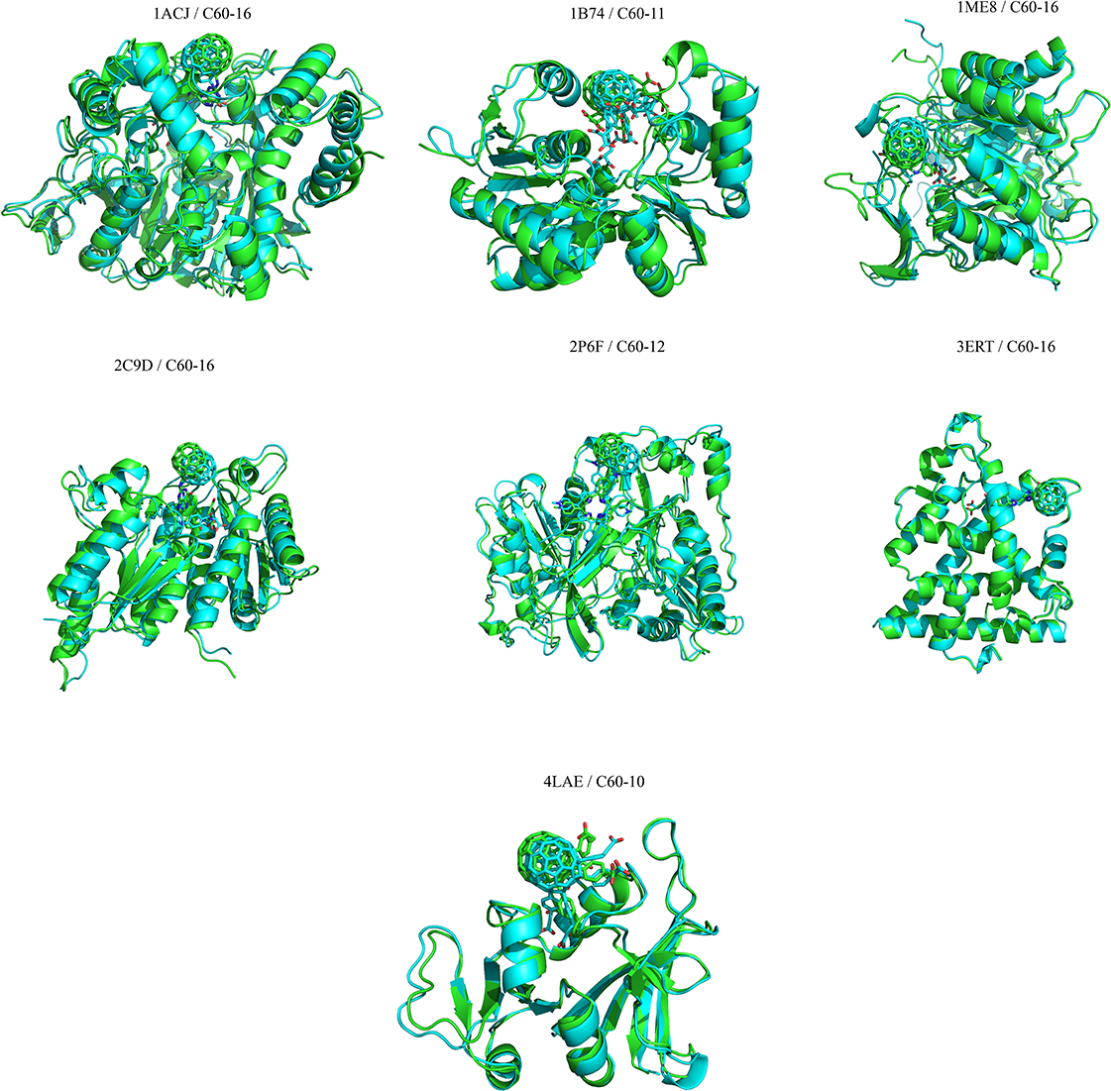
The structural superposition of C60 derivative/protein complexes: The initial structures at time = 0ns and final structures after MD simulation (25ns) for all complexes are superposed. The initial structures are colored in cyan and the final structures are colored in green.

The C60 derivatives from the above-mentioned *in silico* screening were subjected to MD simulation in order to check its stability in the active site of each enzyme. Stability of C60 complexes was measured in term of their Root Mean Square Deviation (RMSD) of Cα from the initial structure. The RMSD of all complexes converged after 15 ns and stayed stable all along the MD trajectory, suggesting that the systems reach equilibrium state after 15 ns. (Fig 8). In general, the RMSD for all the complexes are equal or below the 2 Å suggesting that the binding of C60 derivatives to their respective target does not has significant structural changes during the 25 ns of MD simulation. The structural alignment of initial (0 ns) and final structure (25 ns) for all complexes are in agreement with the RMSD and shows no significant structural changes (Fig 9).

### Conclusion

Since the discovery of C60 molecules in 1980s, the sphere-shaped molecule has found applications in many fields. To explore the potential of this unique molecule further, many water soluble C60 derivatives have been synthesized and their functions have been studied by various groups [32–37]. Although the binding of C60 molecules and their target proteins have been investigated, few studies have been conducted to look into the patterns with which the C60-derivatives could interact with the drug targets. In this study, we have conducted docking and molecular dynamics simulation study of water soluble C60-derivatives with numerous potential drug targets, which include acetylcholinesterase, glutamate racemase, inosine monophosphate dehydrogenase, lumazine synthase, human estrogen receptor alpha, dihydrofolate reductase and N-myristoyltransferase. The docking results show that the C60-derivatives occupy the similar binding site as the native ligands, which support the genuineness of the docking. Interestingly, several water soluble C60-derivatives have better affinities than the native ligands, suggesting they could be better inhibitors for the drug targets. In general, our study provides insights regarding the potential of C60 and C60-derivatives as the therapeutic agents. These results will be instrumental in the development of C60-based medicines against many diseases.

## Supporting Information

S1 Fig Access Tunnel predictions. Location of the access tunnels predicted by the Caver 3.0 for (A) acetylcholinesterase (B) glutamate racemase (C) dihydrofolate reductase (D) N-Myristoyltransferase. The C60 derivatives are represented by spheres and colored in white. The predicted access tunnels are shown in different colors.
(TIF)

S1 Table The two dimensional structures of C60 derivatives. (DOCX)

S2 Table Binding affinities of the FDA approved drugs and C60-16 against acetylcholinesterase. (DOCX)

S3 Table Binding affinities of published inhibitors and C60 derivative against glutamate racemase. (DOCX)
