# Supplementary figures and images for: Analyses of the Binding between Water Soluble C60 Derivatives and Potential Drug Targets through a Molecular Docking Approach
Source: PLoS One. 2016 Feb 1;11(2):e0147761. doi: 10.1371/journal.pone.0147761 (PMC4735121; doi:10.1371/journal.pone.0147761)

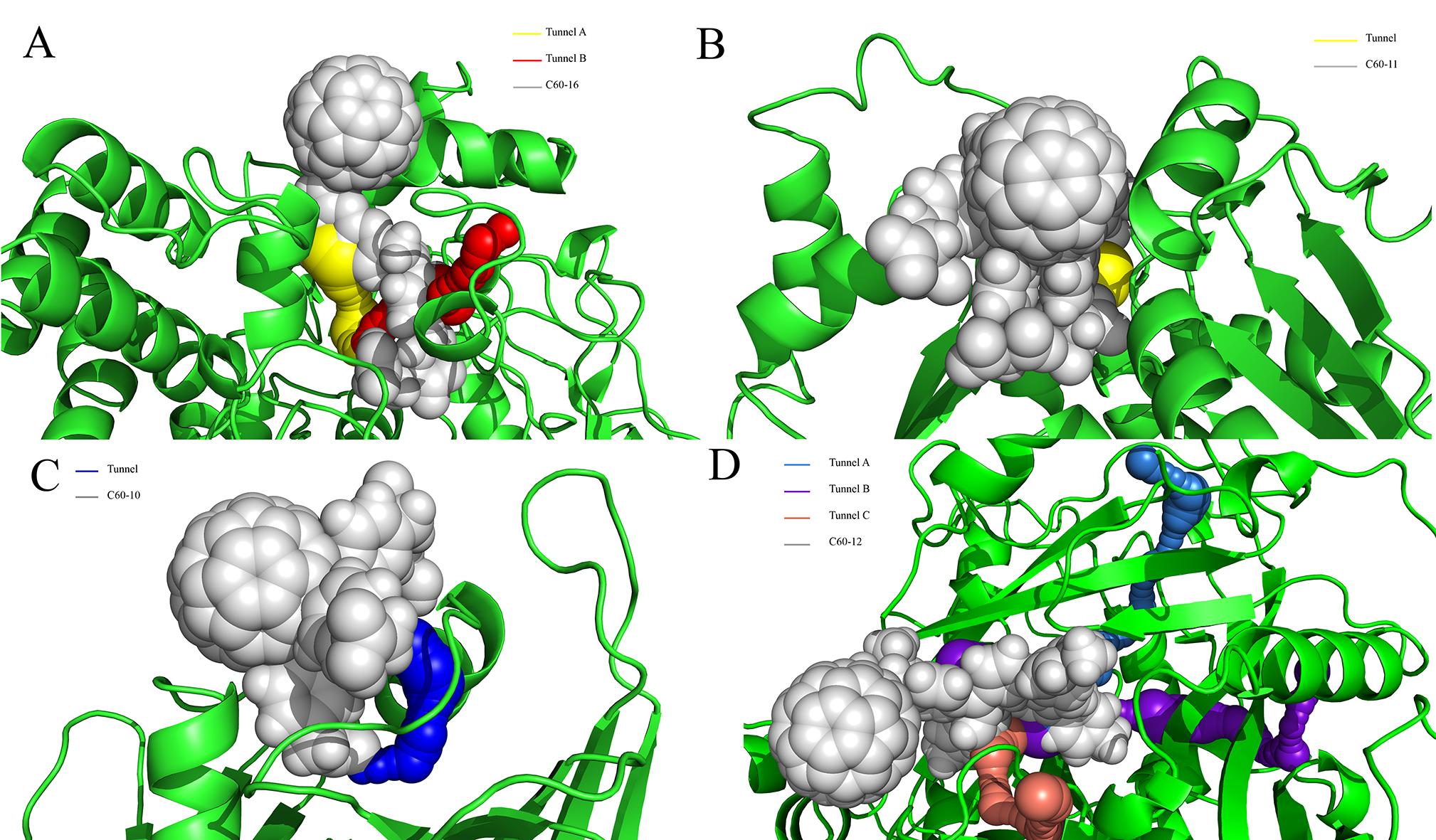

Supplement: S1 Fig — Location of the access tunnels predicted by the Caver 3.0 for (A) acetylcholinesterase (B) glutamate racemase (C) dihydrofolate reductase (D) N-Myristoyltransferase. The C60 derivatives are represented by spheres and colored in white. The predicted access tunnels are shown in different colors. (TIF) [file pone.0147761.s001.tif]
