# Supplementary material for: Analyses of the Binding between Water Soluble C60 Derivatives and Potential Drug Targets through a Molecular Docking Approach
Source: PLoS One. 2016 Feb 1;11(2):e0147761. doi: 10.1371/journal.pone.0147761 (PMC4735121; doi:10.1371/journal.pone.0147761)
Supplement: S1 Table — (DOCX) [file pone.0147761.s002.docx]

Table S1: The two dimensional structure of C60 derivatives. The arc shows the attachment of the C60 molecule

| S.No | Derivative name | Derivative structure | Reference |
| --- | --- | --- | --- |
| 1 | C60-1 | 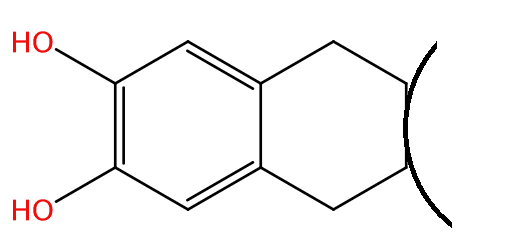 | [[1](#_ENREF_1)] |
| 2 | C60-2 | 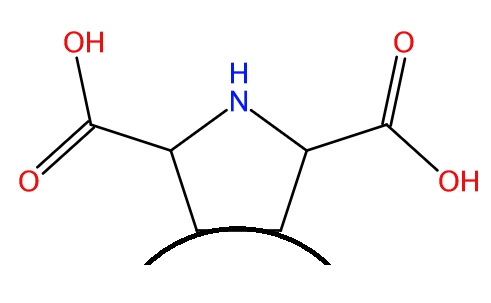 | [[1](#_ENREF_1)] |
| 3 | C60-3 | 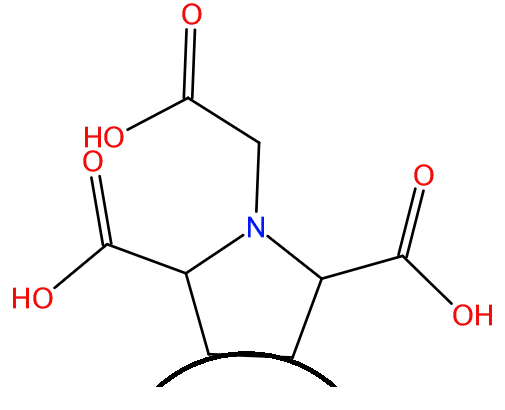 | [[1](#_ENREF_1)] |
| 4 | C60-4 | 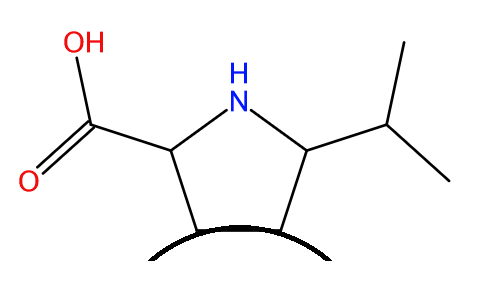 | [[1](#_ENREF_1)] |
| 5 | C60-5 | 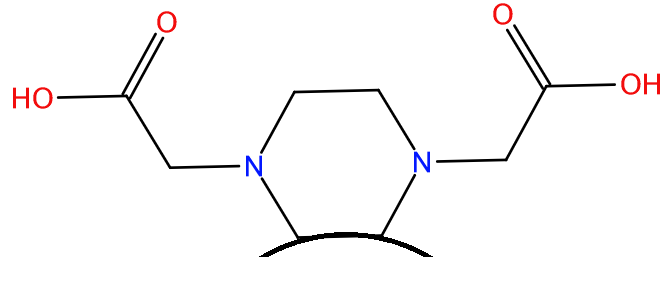 | [[1](#_ENREF_1)] |
| 6 | C60-6 | 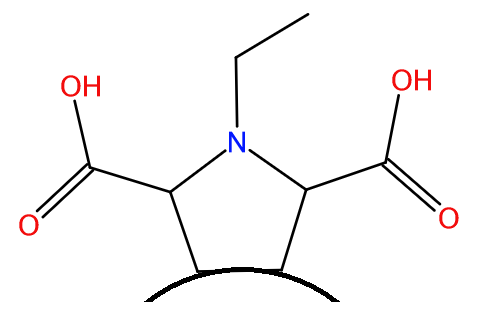 | [[1](#_ENREF_1)] |
| 7 | C60-7 | 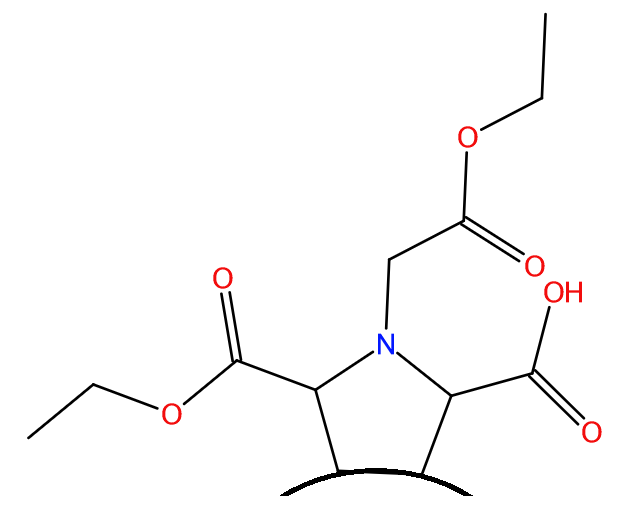 | [[1](#_ENREF_1)] |
| 8 | C60-8 | 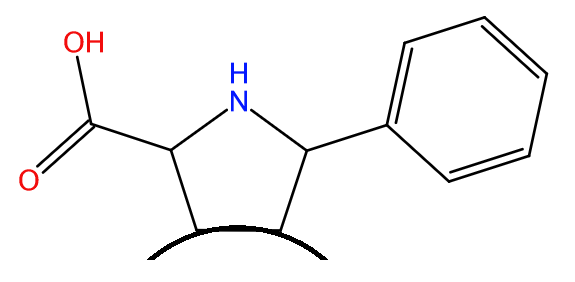 | [[1](#_ENREF_1)] |
| 9 | C60-9 | 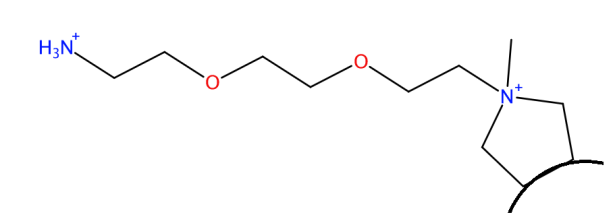 | [[2](#_ENREF_2)] |
| 10 | C60-10 | 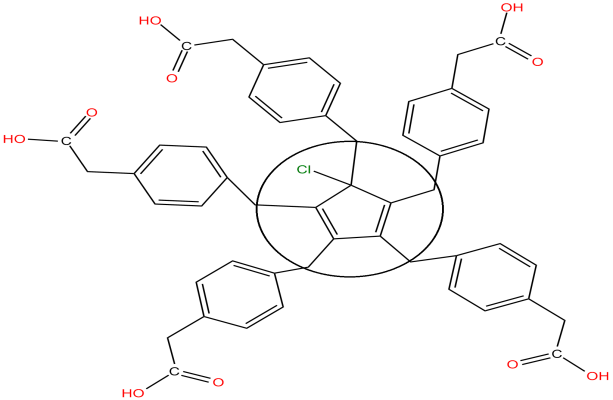 | [[3](#_ENREF_3)] |
| 11 | C60-11 | 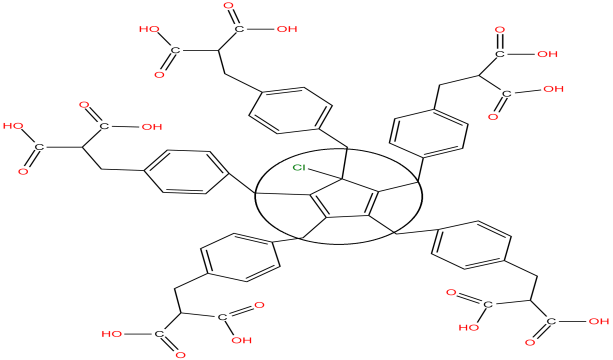 | [[3](#_ENREF_3)] |
| 12 | C60-12 | 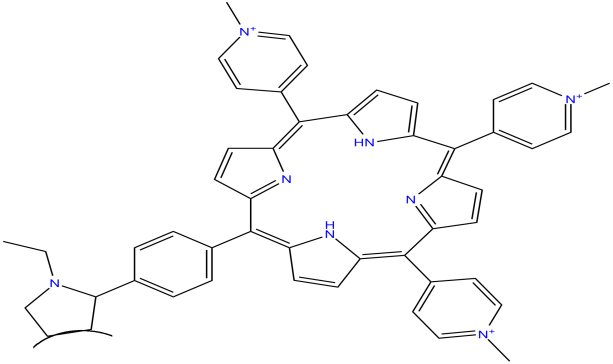 | [[4](#_ENREF_4)] |
| 13 | C60-13 | 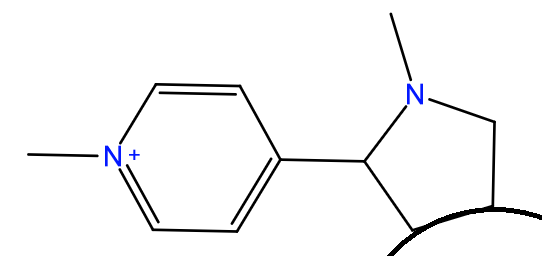 | [[5](#_ENREF_5)] |
| 14 | C60-14 | 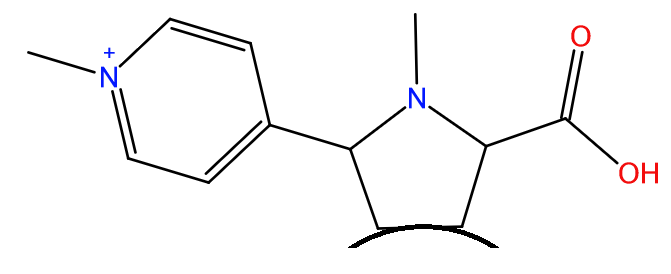 | [[5](#_ENREF_5)] |
| 15 | C60-15 | 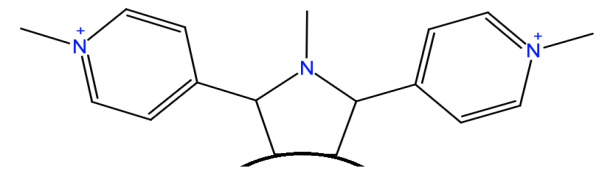 | [[5](#_ENREF_5)] |
| 16 | C60-16 | 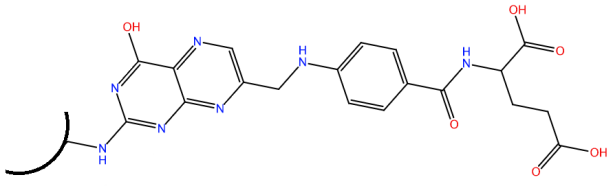 | [[6](#_ENREF_6)] |

1. Shoji M, Takahashi E, Hatakeyama D, Iwai Y, Morita Y, Shirayama R, et al. Anti-influenza activity of c60 fullerene derivatives. PloS one. 2013;8(6):e66337. doi: 10.1371/journal.pone.0066337. PubMed PMID: 23785493; PubMed Central PMCID: PMC3681905.

2. Bosi S, Da Ros T, Spalluto G, Balzarini J, Prato M. Synthesis and anti-HIV properties of new water-soluble bis-functionalized[60]fullerene derivatives. Bioorganic & medicinal chemistry letters. 2003;13(24):4437-40. Epub 2003/12/04. PubMed PMID: 14643341.

3. Troshina OA, Troshin PA, Peregudov AS, Kozlovskiy VI, Balzarini J, Lyubovskaya RN. Chlorofullerene C60Cl6: a precursor for straightforward preparation of highly water-soluble polycarboxylic fullerene derivatives active against HIV. Organic & biomolecular chemistry. 2007;5(17):2783-91. Epub 2007/08/19. doi: 10.1039/b705331b. PubMed PMID: 17700846.

4. Zhou C, Liu Q, Xu W, Wang C, Fang X. A water-soluble C60-porphyrin compound for highly efficient DNA photocleavage. Chemical communications (Cambridge, England). 2011;47(10):2982-4. Epub 2011/01/15. doi: 10.1039/c0cc04919k. PubMed PMID: 21234484.

5. da Silva Goncalves A, Franca TC, Vital de Oliveira O. Computational studies of acetylcholinesterase complexed with fullerene derivatives: a new insight for Alzheimer disease treatment. Journal of biomolecular structure & dynamics. 2015:1-10. doi: 10.1080/07391102.2015.1077345. PubMed PMID: 26219766.

6. Hu Z, Zhang C, Huang Y, Sun S, Guan W, Yao Y. Photodynamic anticancer activities of water-soluble C(60) derivatives and their biological consequences in a HeLa cell line. Chemico-biological interactions. 2012;195(1):86-94. Epub 2011/11/24. doi: 10.1016/j.cbi.2011.11.003. PubMed PMID: 22108244.
