# Supplementary material for: Analyses of the Binding between Water Soluble C60 Derivatives and Potential Drug Targets through a Molecular Docking Approach
Source: PLoS One. 2016 Feb 1;11(2):e0147761. doi: 10.1371/journal.pone.0147761 (PMC4735121; doi:10.1371/journal.pone.0147761)
Supplement: S2 Table — (DOCX) [file pone.0147761.s003.docx]

S2 Table: Binding affinities of the FDA approved drugs and C60-16 against acetylcholinesterase (PDB ID 1ACJ)

| Molecule Name | Pubchem ID | Two dimensional structure | Binding affinity  (kcal/mol) |
| --- | --- | --- | --- |
| Rivastigmine | 77991 | 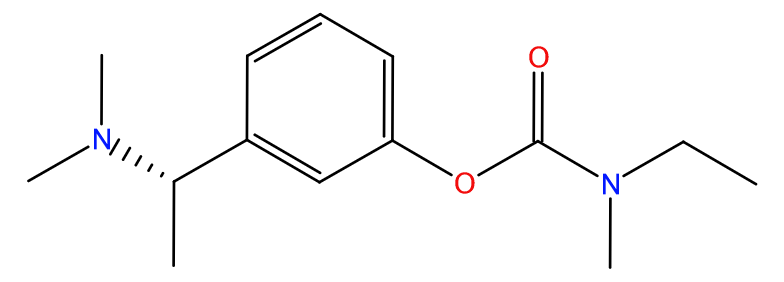 | -7.01 |
| Galantamine | 9651 | 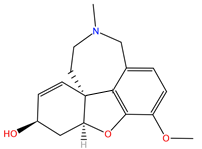 | -7.51 |
| Donepezil | 3152 | 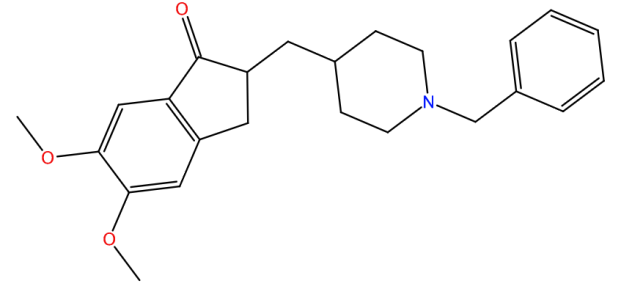 | -8.78 |
| Tacrine | 1935 | 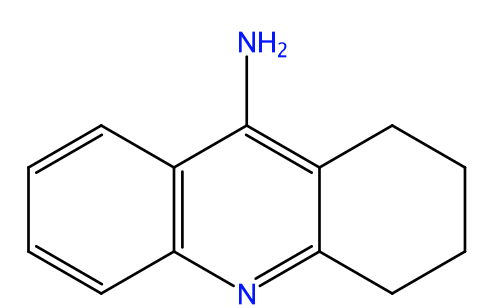 | -6.04 |
| C60-16 |  | 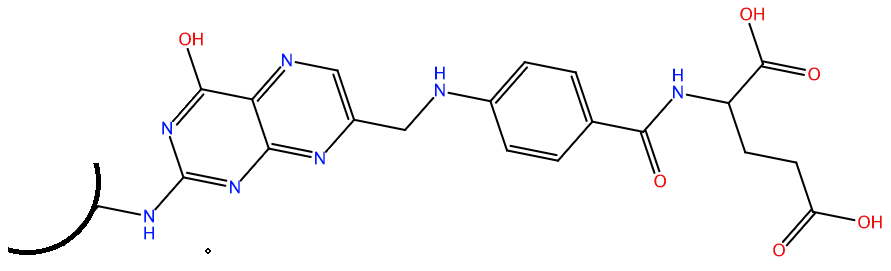 | -14.44 |
