# Supplementary material for: Analyses of the Binding between Water Soluble C60 Derivatives and Potential Drug Targets through a Molecular Docking Approach
Source: PLoS One. 2016 Feb 1;11(2):e0147761. doi: 10.1371/journal.pone.0147761 (PMC4735121; doi:10.1371/journal.pone.0147761)
Supplement: S3 Table — (DOCX) [file pone.0147761.s004.docx]

S3 Table: Binding affinities of published inhibitors and C60 derivative against glutamate racemase (PDB ID 1B74)

| Name | Molecule structure | Binding affinity  (kcal/mol) |
| --- | --- | --- |
| C1 | 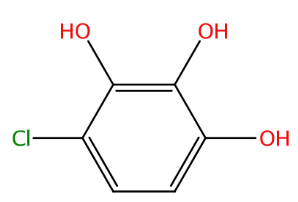 | -4.049 |
| C2 | 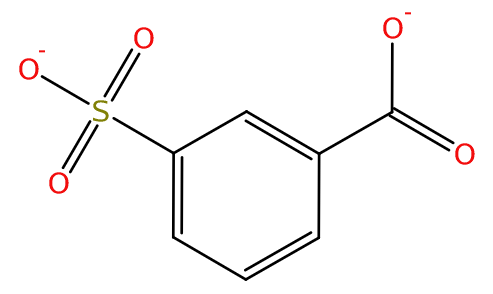 | -3.45 |
| C3 | 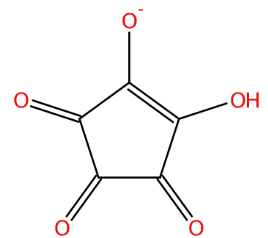 | -2.39 |
| C4 | 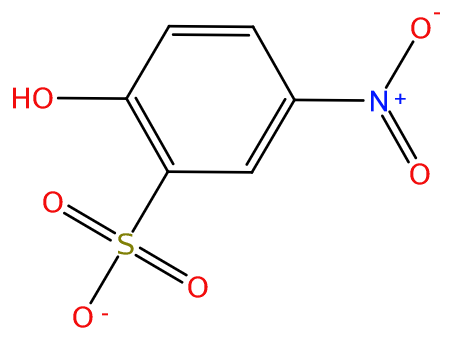 | -3.63 |
| C5 | 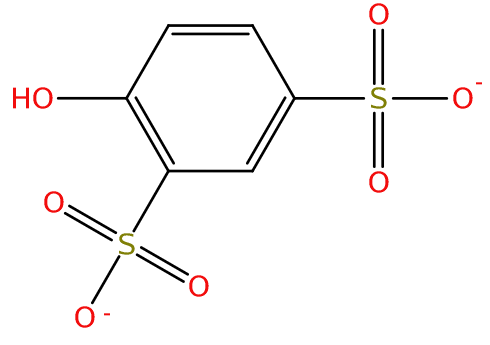 | -3.83 |
| C6 | 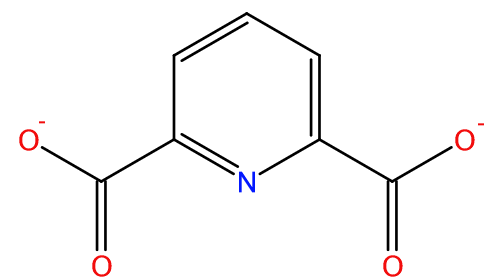 | -3.53 |
| C7 | 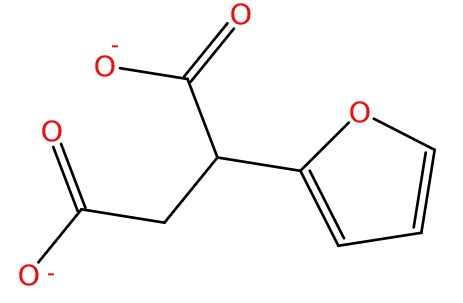 | -3.03 |
| C8 | 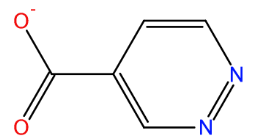 | -2.74 |
| C60-11  (C60 derivative) | 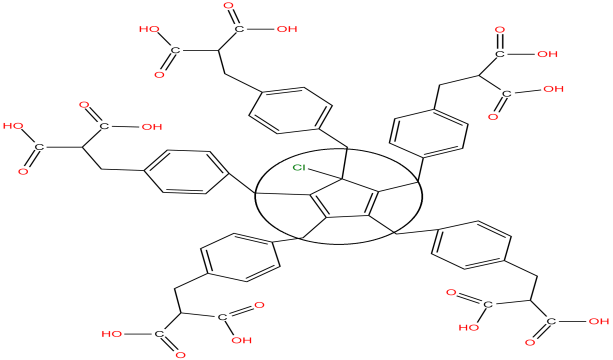 | -13.79 |
| D-glutamine (substrate analogue) | **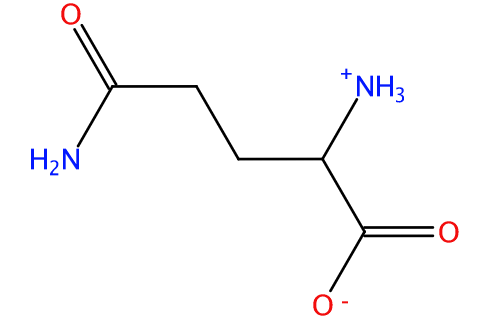** | -3.19 |
